# Exploring the Astrovirome of Shellfish Matrices Using Nanopore Sequencing

**DOI:** 10.3390/vetsci10030175

**Published:** 2023-02-21

**Authors:** Farzad Beikpour, Francesco Pellegrini, Gianvito Lanave, Michele Camero, Cristiana Catella, Barbara Di Martino, Federica Di Profio, Chiara Masotti, Roberta Battistini, Laura Serracca, Giuseppina La Rosa, Vito Martella, Elisabetta Suffredini

**Affiliations:** 1 Department of Veterinary Medicine, University of Bari Aldo Moro, 70010 Valenzano, Italy; 2 Faculty of Veterinary Sciences, University of Teramo, Località Piano d’Accio, 64100 Teramo, Italy; 3 Department of La Spezia, Istituto Zooprofilattico Sperimentale del Piemonte, Liguria e Valle d’Aosta, Via degli Stagnoni 96, 19100 La Spezia, Italy; 4 Department of Environment and Health, Istituto Superiore di Sanità, 00161 Rome, Italy; 5 Department of Food Safety, Nutrition and Veterinary Public Health, Istituto Superiore di Sanità, Viale Regina Elena 299, 00161 Rome, Italy

**Keywords:** astrovirus, shellfish, virome, enteric

## Abstract

**Simple Summary:**

Astroviruses are important human pathogens, associated with gastro-enteric disease in children and recently with encephalitis in immunocompromised patients. Astroviruses have also been identified in mammals, birds, lower vertebrates and invertebrates in association with enteric and extra-intestinal diseases or, in some cases, as components of the enteric virome without a clear link with specific clinical signs. As a proof of concept, we conjugated the versatility and broad reactivity of a commonly used consensus primer set, able to amplify in a nested RT-PCR protocol the RNA-dependent RNA polymerase (RdRp) of most members of the *Astroviridae* family, with a nanopore sequencing platform, in order to assess the potential of this approach to generate astroviromic data in complex matrices. Amplicons generated from mussels were used to generate libraries, either alone or in pools, and subjected to deep sequencing. Overall, we identified a variety of known and unknown RdRp sequence types, in most cases distantly related to astrovirus sequences available in the databases. Avian-origin astrovirus sequences were predominant, likely due to contamination of surface water.

**Abstract:**

Astroviruses are important human enteric pathogens transmissible with contaminated food and water. Astroviruses have also been identified in mammals, birds, lower vertebrates and invertebrates. The genetic diversity of human and animal astroviruses poses a challenge for diagnostics and taxonomy. As a proof of concept, we used a panastrovirus consensus primer set, able to amplify in a nested RT-PCR protocol a 400-nt-long fragment of the RNA-dependent RNA polymerase of most members of the *Astroviridae* family, in conjunction with a nanopore sequencing platform, to generate information on the astrovirome in filter-feeding mollusks. Amplicons generated from bivalve samples were used to generate libraries for deep sequencing. In three samples, only one unique RdRp sequence type was obtained. However, in seven samples and in three barcodes with eleven pooled samples, we identified a variety of known and unknown RdRp sequence types, in most cases distantly related to astrovirus sequences available in the databases. In total, 37 different sequence contigs were generated. Avian-origin astrovirus sequences were predominant, likely due to contamination of shellfish harvesting waters by marine birds. Astroviruses of the aquatic eco-system were also identified, whereas human astroviruses were not detected.

## 1. Introduction

Astroviruses (AstVs), family *Astroviridae*, are a group of small, non-enveloped viruses with a single-strand genomic RNA of positive polarity and an icosahedral capsid of 27–30 nm in diameter [1]. The genome length is 6.8 to 7.9 kb and includes three open reading frames (ORFs), namely ORF1a, ORF1b and ORF2. ORF1a and ORF1b encode nonstructural proteins, a serine protease, and an RNA-dependent RNA polymerase (RdRp), whereas ORF2 encodes the viral capsid protein [1]. AstVs have been classified by the International Committee on Taxonomy of Viruses (ICTV) into two genera, namely *Mamastrovirus* (MAstV) and *Avastrovirus* (AAstV), known to infect mammals and birds, respectively. Since the first description of human AstV in children with diarrhea in 1975 [2], an impressive number of AstVs have been reported in several animal species including mammals, birds, reptiles, amphibians, fish and even invertebrates [3,4,5,6,7], posing a challenge for their classification under the genus level [8,9].

Human AstVs are responsible for acute gastroenteritis (AGE) with worldwide distribution, chiefly in pediatric populations [1]. AstVs have also been identified in adult and elderly populations and associated with large foodborne outbreaks [1]. Human infections are predominantly caused by the mamastrovirus species 1 (MAstV-1), also referred to as “classic” human AstVs [1]. In the last decade, genetically divergent human AstV species, referred to as “atypical” or “animal-like” strains, have been discovered, including strain Melbourne (MLB) (MAstV-6) [10], strains Virginia/Human-Mink-Ovine-like (VA/HMO) (MAstV-8 and MAstV-9) [10,11,12] and the tentative species MAstV-20 [13]. The role of animal-like HAstVs as enteric pathogens is uncertain [14]. Both classical and atypical HAstVs have been associated with either respiratory illnesses [15] or central nervous system infections in vulnerable subjects [14,16,17,18,19].

Broadly reactive consensus PCR protocols for panviral amplification at family, subfamily or genus levels is a straightforward approach used for virus discovery and for epidemiological studies based on screening of large sample collections. To improve the sensitivity of PCRs using consensus/degenerated primers, panviral PCR protocols have often been designed using a nested or heminested strategy [16,20,21,22]. The diversity of AstVs in humans and animals also poses several challenges for diagnostics. A panastrovirus RT-PCR protocol was first described in 2008 [23] to investigate the circulation of AstVs in bats and subsequently largely adopted and, since then, it has been cited/used in nearly two hundred studies. This protocol amplifies a 422-nt-long portion of the RdRp (ORF1b) with a nested PCR strategy and it has been successfully applied to generate information from several animal hosts.

Filter-feeding mollusks are able to concentrate viruses in their tissues from waters, and therefore they may be considered as bio-indicator animals [24]. Since mollusks are often consumed raw, they are considered a common source of exposure for human populations. Accordingly, they represent a valuable target to use to explore the diversity of the AstV population, or “astrovirome”. As a proof of concept, we applied the panastrovirus protocol to shellfish samples, since consumption of raw mollusks (mussels, clams, oysters) represents an important source of infection for human populations with enteric (rotavirus, norovirus, astrovirus) and hepatotropic viruses (hepatitis A and E) [25,26,27]. We therefore coupled AstV-targeted RT-PCR enrichment with a deep-sequencing Nanopore platform to assess the potential of an astroviromic implementation in food or environmental virology. This approach, based on the targeted, nested RT-PCR enrichment of the viral genome, is indeed expected to exceed the sensitivity of metagenomic protocols, in which unbiased enrichment of nucleic acids of eukaryotic and prokaryotic organisms tends to overwhelm and mask the virome component.

## 2. Materials and Methods

### 2.1. Collection of Samples

Samples were collected within two sampling campaigns:

(a) Oyster (*Crassostrea gigas*) sampling within the “European baseline survey of norovirus in oysters” [28,29] (November 2016–October 2018). Samples were taken every two months from four production areas (one class A and three class B, according to the EU Commission Implementing Regulation 2019/627) located in the northern and southern Adriatic and Thyrrenian sea, respectively (Venetian Lagoon, Apulia, Gulf of La Spezia and Sardinia). A total of 48 oyster samples were collected.
(b) Bivalve shellfish (*Mytilus galloprovincialis*, *Crassostrea gigas*, *Ostrea edulis*) sampling in the Gulf of La Spezia (December 2020–December 2021). Samples were taken monthly from nine production areas (two class A and seven class B). A total of 86 samples was collected (*Mytilus* n = 43, *Crassostrea* n = 37, *Ostrea* n = 6).

### 2.2. RNA Extraction

Viral recovery from bivalve mollusks was carried out as reported using the ISO 15216-1:2017 method (ISO 15216-1:2017). Briefly, digestive tissue was removed by dissection from each bivalve, pooled, and finely chopped. Aliquots of 2 g were spiked with 10 μL of process control virus (Mengovirus), digested with 2 mL of proteinase K (0.1 mg/mL) at 37 °C for 60 min with shaking (320 rpm), and then treated at 60 °C for 15 min to inactivate the enzyme. Then, samples were centrifuged at 3000× *g* for 5 min, supernatants were collected, and volumes recorded. Viral RNA was extracted using NucliSENS^®^ magnetic extraction reagents (bioMerieux, Marcy l’Etoile, France) according to the manufacturer’s instructions from 500 μL of the supernatants. Finally, RNA was eluted (100 μL) and used immediately for RT-PCR analysis or stored at −80 °C.

### 2.3. RT-PCR Screening

Screening for AstVs was conducted using a nested reverse transcription (RT)-PCR protocol with a broadly reactive set (panastrovirus) of primers targeting the RdRp region and amplifying the majority of human and animal AstVs [23]. For reverse transcription of RNA and PCR amplification, SuperScript IV One-Step RT-PCR kit (Invitrogen, Life Technologies, Waltham, MA, USA) was used with primers PanAV-F11 (GARTTYGATTGGRCKCGKTAYGA) and PanAV-F12 (GARTTYGATTGGRCKAGGTAYGA), and reverse primer PanAV-R1 (GGYTTKACCCACATNCCRAA).

Two microliters of the product of first-round amplification were diluted 1:100 and used as a template for the second-round amplification, using DreamTaq polymerase (Thermo Fisher Scientific, Waltham, MA, USA) with primers PanAV-F21 (CGKTAYGATGGKACKATHCC) and PanAV-F22 (AGGTAYGATGGKACKATNCC) and the same reverse primer, PanAV-R1. The PCR products were run on a 1.5% agarose gel containing a fluorescent nucleic acid marker (GelRed^®^ Nucleic Acid Gel Stain; Biotium, Fremont, CA, USA) at 90 V for 50 min and visualized under fluorescent light on the Gel Doc imaging system (Bio-Rad Laboratories, Hercules, CA, USA). AstV-specific amplicons of about 420 nt in length were visualized and excised from gel for purification with the GRS PCR&Gel purification kit (Grisp, Porto, Portugal).

### 2.4. Oxford Nanopore Technologies (ONT) Sequencing and Genome Detective Pipeline

Amplicons were selected based on visualization on gel electrophoresis and were quantified using a Qubit dsDNA HS kit (Invitrogen, Life Technologies, Milan, Italy). The Rapid Barcoding Kit 24 SQK-RBK114.24 (Oxford Nanopore Technologies, ONT^TM^, Oxford, UK) was used to prepare libraries, which were purified using Agencourt AMPure XP magnetic beads (Beckman Coulter™). The libraries were pooled and sequenced using MinION Flow Cell (R9.4.1) FLO-FLG001 on the MinION- Mk1C device (ONT^TM^, UK) with a Flongle adapter for 24 h.

The reads were analyzed using the web-based bioinformatics service Genome Detective Virus Tool v 2.48 (GDVT) [30]. In the GDTV pipeline the reads were trimmed to remove adaptors and quality-filtered with a Trimmomatic [31]. Candidate viral reads were identified using the protein-based alignment method DIAMOND [32] and sorted in buckets. Therefore, the reads were de novo assembled using SPAdes [33]. The contigs were analysed using Blastx and Blastn to query the NCBI RefSeq virus database and joined together using the Advanced Genome Aligner (AGA) [34].

### 2.5. Sequence and Phylogenetic Analyses

The online tool FASTA (http://www.ebi.ac.uk/fasta33, accessed on 12 January 2023) was employed using the default values to find homologous hits. Sequence editing and multiple codon-based (translation) alignments were performed by Geneious Prime v. 2021.2 (Biomatters Ltd., Auckland, New Zealand). The sequences were aligned with cognate AstVs retrieved from the GenBank database by MAFFT [35]. The correct substitution model settings for the phylogenetic analysis and estimation of selection pressure on coding sequences were derived using “Find the best protein DNA/Protein Models” implemented in MEGA X v. 10.0.5 software [36]. The evolutionary history was inferred by using the maximum-likelihood method, the Tamura-Nei 4-parameter model, a discrete gamma distribution and invariant sites to model evolutionary rate differences among sites (6 categories) and supplying statistical support with 1000 replicates. Bayesian inference and neighbor-joining approaches were also used to explore the phylogeny of AstVs.

## 3. Results

### 3.1. RT-PCR Screening

The RT-nested PCR screening yielded amplification in 3 of the 48 (6.3%) oyster samples collected in the 2016–2018 sampling campaign. These positive samples had been all collected—in December 2016, July 2017 and February 2018, respectively—from the class B production area located in the Gulf of La Spezia. Among the 86 bivalve samples collected between 2020 and 2021 in the Gulf of La Spezia, 18 (20.9%) provided an amplicon of the expected size. Amplification was achieved in both mussel (n = 9) and oyster (n = 9) samples and included samples collected from all the nine production areas included in the study.

### 3.2. ONT Sequencing

We tested 9 samples with a visible band in gel electrophoresis and 12 samples with a faint amplicon close to the expected size. A total of 11 samples with a weak amplicon were pooled (3 or 4 samples per pool) before library preparation, whilst one sample (BR13) was tested apart. The DNA in the samples ranged from 0.509 to 12.5 ng/µL.

**Figure 1:**
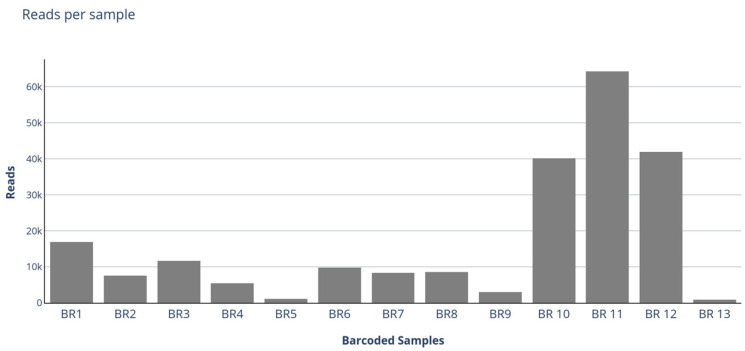
Histogram with the read number per sample. BR7, BR8 and BR9 each contained 3 or 4 pooled samples (11 samples in total).

A total of 340.370 Mb of data was produced, resulting in 491.410 reads. The GDTV pipeline identified 219,564 (44.6%) AstV-specific reads out of 491,410 reads produced by the run. The number of AstV reads per sample ranged from 845 to 64,214 (median = 8533) (Figure 1).

**Figure 2:**
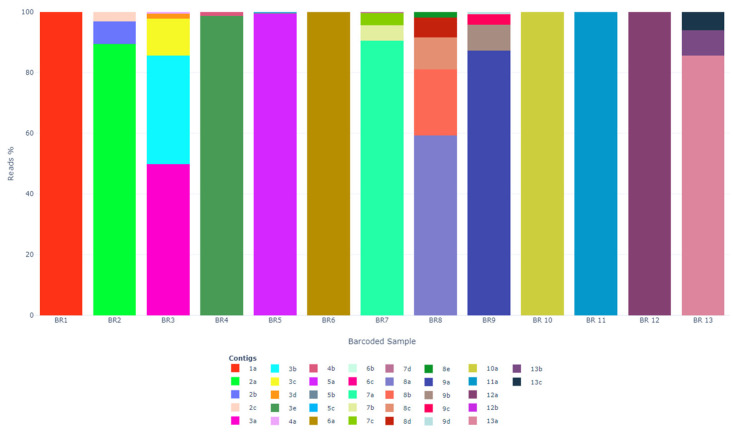
Bar chart showing reads per sequence type’s composition in each barcode (BR). BR7, BR8 and BR9 each contained 3 or 4 pooled samples (11 samples in total).

A total of 37 AstV RdRp sequence contigs were generated by GDTV. The number of reads per contig ranged from 1 to 64,214 (mean 4958, median 561). Even contigs with low coverage were retained in the analysis. For each barcode, the proportion of reads per sequence type is provided in Figure 2.

Overall, a mean of 2.8 contigs per sample (range 1–5) was obtained. The qualitative and quantitative information obtained for the different samples is summarized in Table 1.

In three samples (BR1, BR10 and BR11), only a unique RdRp sequence type was obtained. However, in six samples and in the pooled samples (BR7, BR8 and BR9), we identified a variety of known and unknown RdRp sequence types, in most cases distantly related to AstV sequences available in the databases (Table 1). Multiple AstV sequence types were also retrieved in the BR13 sample. Avian-like AstV sequences were predominant (23/37, 62.1%), whereas two bat-like AstV sequences (#4b, #9d) were identified (5.4%). Two RdRp sequences (#7d and #8c) (5.4%) displayed the highest nt identity to the reptilian host. A group of RdRp sequence types (10/37, 27.1%) was likely derived from the marine eco-system, distantly related to AstV sequences from lower vertebrates and invertebrates of aquatic ecosystems.

### 3.3. Phylogenetic Analysis

**Figure 3:**
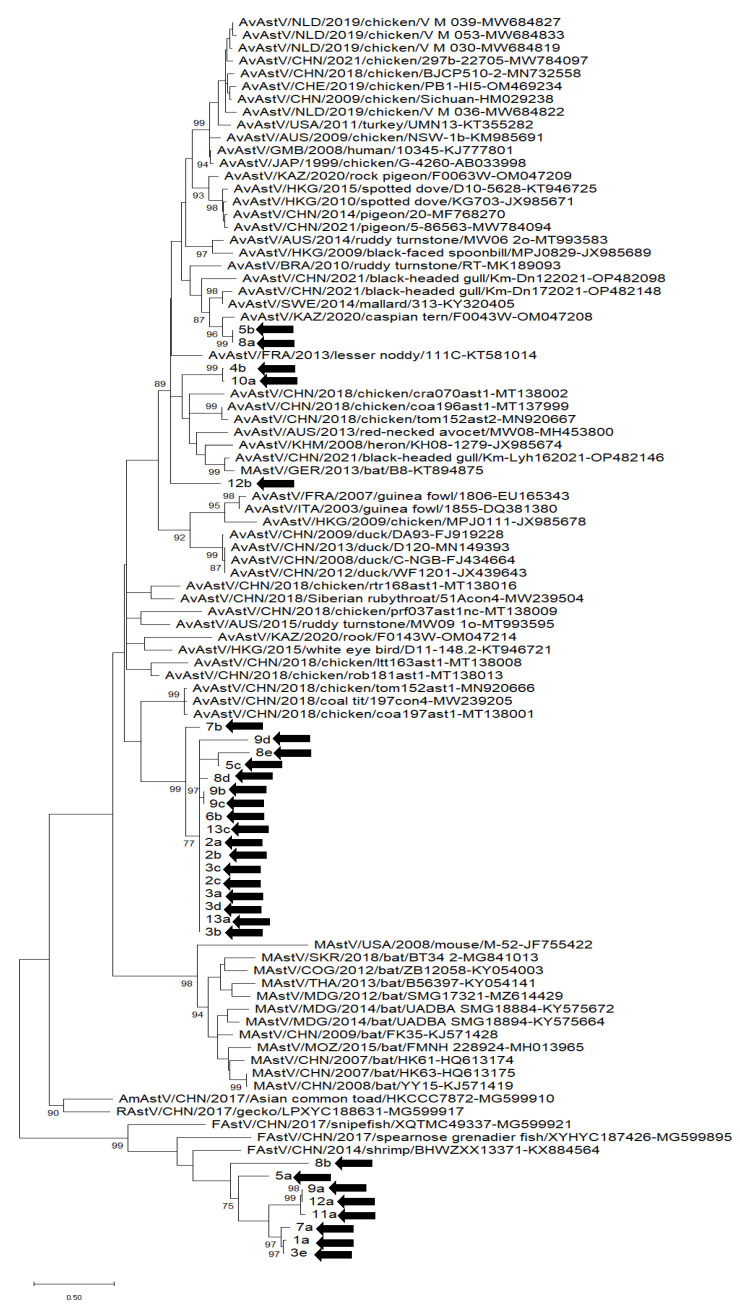
Unrooted phylogenetic tree based on partial ORF1b (201nt) of Astrovirus (AstV) strains identified in this study and reference strains recovered in the GenBank database. AstV strains 4a, 6a, 6c, 7c, 7d, 8c and 13b were excluded from the phylogenetic tree due to the limited size of the sequences obtained. The Maximum Likelihood method and Tamura-Nei model (four parameters) with a gamma distribution and invariable sites were used for the phylogeny. One thousand bootstrap replicates were used to estimate the robustness of the individual nodes on the phylogenetic tree. Bootstrap values higher than 75% are displayed. Black arrows mark strains detected in this study. Numbers of nucleotide substitutions are indicated by the scale bar. MastV = AstV identified in mammalian host; AAstV = AstV identified in avian host; RastV = AstV identified in reptilian host, AmAstV identified in amphibian host; FastV = AstV identified in fish and marine animals.

A phylogenetic analysis based on a partial ORF1b sequence (201 nt) of AstVs was carried out using the sequence contigs generated in the study and the closest relatives identified in the GenBank database. Different algorithms were used to explore the phylogeny of the AstVs, and similar topologies with few differences in bootstrap values at the nodes of the tree were observed. Accordingly, the maximum-likelihood tree was retained. Overall, we identified three major groups of sequences. Two groups comprised almost exclusively avian-related sequences. A third group exclusively encompassed sequence types either closely or distantly related to AstV sequences retrieved from animals of the marine ecosystem (Figure 3).

## 4. Discussion

In this study we coupled a consolidated panastrovirus protocol [23] with a deep-sequencing ONT^TM^ platform and applied this strategy to shellfish samples, to assess the potential of an astroviromic implementation in food or environmental virology. This approach, based on targeted nested RT-PCR enrichment of astroviruses, is indeed expected to exceed the sensitivity of metaviromic protocols, since unbiased enrichment of nucleic acids of eukaryotic and prokaryotic organisms tends to mask the virome component. To overcome this problem, which could hinder virome investigations or viral genome sequencing, several approaches have been attempted; for instance, trying to optimize the procedures for extraction of viral nucleic acids [37], selecting the viral target with pools of oligo baits [38] or performing a PCR enrichment with specific primers [39]. A further advantage of this strategy is that when testing filter-feeding animals such as bivalve shellfish, the amplicons generated with a panviral PCR protocol likely consist of a mixed population of virus sequences and are, in most cases, not suitable for direct Sanger sequencing. At the same time, a limit of this astroviromic approach is the possible selection of some AstV templates over others due to primer design and the two-step amplification PCR. This bias has been noted in some experiments based on consensus primers for noroviruses [40].

Using our astroviromic approach, we were able to generate 37 AstV sequence contigs from 13 barcodes encompassing 21 shellfish samples (with 11 samples pooled in 3 barcodes), with a mean of 2.8 contigs per barcode (range 1–5). Although some contigs were constructed using only a low number (≤6) of reads (#5b, 5c, 6b and 6c), they were retained in our analysis since we did not apply a cutoff and were sticking to the raw results of the GD software pipeline.

Only 5/37 (13.5%) sequences scored more than 80.1% nt identity to sequences available in the databases using FASTA, whereas 2/37 (5.4%) sequences scored between 72.2 and 80.0% nt identity, and 30/37 (81.1%) scored between 57.6 and 69.3% (Table 1). Accordingly, the majority of the RdRp sequence types (32/37, 86.5%) could not be assigned firmly, as sequence identity was lower than 69.3% nt to RdRp sequences available in the database, hinting at the massive and overwhelming genetic diversity of this viral family.

Overall, the RdRp sequence types could be classified into two major groups in sequence and phylogenetic analysis (Figure 1). A large group included n = 10 AstV sequences distantly related to sequences associated with animals of the marine ecosystem, including shrimp and fish [3,4]. The possibility that some of the generated contigs were actually mollusk-associated AstVs, rather than viruses of other marine animal sources accumulated passively during water filtration for feeding by the mussels, should not be ruled out, since AstV RNA has been identified in lophotrochozoans [3].

In the last decade, the adoption of massive sequencing technologies has uncovered novel putative AstV species from humans and from different mammalian and avian hosts, providing an unprecedented challenge for diagnostics and for taxonomical classification of these viruses [9]. Metatranscriptomic investigations have also discovered AstVs in invertebrate and lower vertebrate animals [3,4,6]. Metagenomic analysis of invertebrate animals from land, fresh, coast and marine waters from China has identified AstV RNA in crustaceans, myriapods and lophotrochozoans, revealing the magnitude of the genetic diversity of RNA viruses in these animals [3]. Likewise, a large metatranscriptomic investigation for RNA viruses in lower vertebrates, including species of the classes *Agnatha* (jawless fish), *Chondrichthyes* (cartilaginous fish), *Actinopterygii* (ray-finned fish), *Amphibia* (frogs, salamanders and caecilians) and *Reptilia* (snakes, lizards and turtles) has identified AstV sequences in the gill, liver, lung and gut of several animals, whereas AstV RNA was not identified from the classes *Leptocardii* (lancelets) and *Sarcopterygii* (lungfish) [4]. Importantly, both the studies indicate that AstVs are a common component of the virome of aquatic animals.

In our analysis, a large group of contigs encompassed 27 RdRp sequences, mostly of AAstVs or avian-like AstVs (n = 23), and to a lesser extent of bat-like (n = 2) and reptilian AstVs (n = 2). AAstVs have been associated with a broad spectrum of clinical signs, including enteritis in turkeys, chickens and guineafowl, mild growth depression and nephritis in chickens, and hepatitis in ducklings [41]. Also, AAstVs have been associated with pre-hatching mortality in ducklings and goslings [42]. In our analysis, contigs #5b, #6c, #7c, #8a and #13b were closely related (80.1–92.5% nt identity) to AAstV-2 mallard strain SWE/2014/313, and contig #17 was related (75% nt identity) to a chicken AAstV, whereas most contigs were more distantly related (<70% nt identity) to other AAstVs. Metagenomic investigations in different avian species have revealed that AAstVs are genetically highly diverse [8,43]. The abundance and diversity of AAstV sequences in the shellfish samples tested in our study likely reflects fecal contamination of waters by birds living in the coastal areas, since shellfish production areas are frequently located close to the coastline, and are ideal habitats for several aquatic birds which may also feed on bivalves emerging from the water surface. A few (n = 2, 5.4%) sequences (#4b, #9d) were related to AstV sequences retrieved in bats (68.4–72.2%). Likewise, a few (n = 2) sequences (#7d, #8c) were related (67.9–69.3) to reptilian AstVs.

No human AstV sequence was generated in our experiments, although targeted screening by quantitative RT-PCR of the samples tested in our experiment detected human AstV in two mussel samples pooled in barcode BR9, but only with a very low concentration (*Cq* > 38.5) (Battistini, *unpublished*). This could be explained by the low concentration of human AstVs, consistent with the expected low level of human fecal contamination in high quality bivalve production waters (class A and B). Also, the abundance of AstVs of aquatic and avian origin likely masked the presence of human AstVs, saturating the broadly-reactive amplification of our astroviromic protocol.

Testing the astroviromic protocol with samples collected from areas with lower microbiological standards (class C areas) or environmental samples from wastewater treatment plants could help provide better understanding of the potential of this approach.

## 5. Conclusions

In conclusion, as a proof of concept, we assessed the potential of a commonly used panastrovirus protocol in combination with a deep sequencing Nanopore platform to explore the diversity of the astrovirome in shellfish samples. The protocol was able to identify 2.8 RdRp sequence contig types per tested barcode, and the majority of AstV sequences reflected water contamination by birds, by animals of the aquatic ecosystem or, to a lesser extent, by bats and reptiles. Interestingly, AAstVs related to avian nephritis viruses 1 and 2 have been detected in African children [13], and serological studies have detected antibodies to turkey astrovirus type 2 in up to 26% of poultry workers [44], suggesting that some AAstVs may be transmitted to humans. The possibility of interspecies birds-to-mammals transmission has also been documented in minks [45]. Since seafood products may occasionally be consumed raw or undercooked, our findings suggest caution towards the potential, yet remote, risk of viral transmission. Since AstVs are common components of the enteric virome of several animals, the possibility of using astroviromic data as a proxy for the microbiological quality of water and food should be further explored.

## Figures and Tables

**Table 1:** Results of sequence analysis on the contigs generated with the astroviromic protocol. The number of reads per contig, the depth of coverage for each contig and the best match upon FASTA interrogation of the Ensemble database of the European Bioinformatics Institute (accessed on 31 December 2022) are shown. Classification at the genus level is based on NCBI taxonomical assignment.

Barcode	Nr Contig	Reads	Depth of Coverage	Identity to Reference Strains			
				Genus	Accession nr	FASTA Nucleotide	nt identity %
BR1	1a	16,846	1870	Unclassified	KX884564	FAstV/CHN/2014/shrimp/BHWZXX13371	60.1
BR2	2a	6733	5255.6	Avastrovirus	KT946721	AAstV/HKG/2015/white eye bird/D11-148.2	64.4
	2b	566	482.6	Avastrovirus	KT946721	AAstV/HKG/2015/white eye bird/D11-148.2	63.5
	2c	228	221.4	Avastrovirus	KT946721	AAstV/HKG/2015/white eye bird/D11-148.2	63.8
BR3	3a	5831	489.8	Avastrovirus	KT946721	AAstV/HKG/2015/white eye bird/D11-148.2	64.3
	3b	4208	3625.2	Avastrovirus	KT946721	AAstV/HKG/2015/white eye bird/D11-148.2	64.1
	3c	1413	1151.6	Avastrovirus	KT946721	AAstV/HKG/2015/white eye bird/D11-148.2	64.6
	3d	208	235.3	Avastrovirus	KT946721	AAstV/HKG/2015/white eye bird/D11-148.2	64.9
	3e	57	51.6	Unclassified	KX884564	FAstV/CHN/2014/shrimp/BHWZXX13371	58.0
BR4	4a	5417	13,488.9	Unclassified	MG599895	FAstV/CHN/2017/spearnose grenadier fish/XYHYC187426	60.2
	4b	67	142.8	Mamastrovirus	KT894875	MAstV/GER/2013/bat/B8	72.2
BR5	5a	1147	1474.7	Unclassified	KX884564	FAstV/CHN/2014/shrimp/BHWZXX13371	59.9
	5b	3	5.9	Avastrovirus	KY320405	AAstV/SWE/2014/mallard/313	81.9
	5c	3	4.9	Avastrovirus	JX985678	AAstV/HKG/2009/chicken/MPJ0111	63.7
BR6	6a	9727	19,702.3	Unclassified	KX884564	FAstV/CHN/2014/shrimp/BHWZXX13371	59.7
	6b	5	6	Avastrovirus	KT946721	AAstV/HKG/2015/white eye bird/D11-148.2	62.2
	6c	1	2	Avastrovirus	KY320405	AAstV/SWE/2014/mallard/313	80.1
BR7	7a	7566	809	Unclassified	KX884564	FAstV/CHN/2014/shrimp/BHWZXX13371	57.6
	7b	425	450.3	Avastrovirus	KT946721	AAstV/HKG/2015/white eye bird/D11-148.2	67.5
	7c	326	669.4	Avastrovirus	KY320405	AAstV/SWE/2014/mallard/313	92.5
	7d	31	59.4	Unclassified	MG599917	RAstV/CHN/2017/gecko/LPXYC188631	69.3
BR8	8a	5065	540.5	Avastrovirus	KY320405	AAstV/SWE/2014/mallard/313	82.2
	8b	1861	1861	Unclassified	KX884564	FAstV/CHN/2014/shrimp/BHWZXX13371	59.9
	8c	897	1224.9	Unclassified	MG599917	RAstV/CHN/2017/gecko/LPXYC188631	67.9
	8d	561	474	Avastrovirus	KT946721	AAstV/HKG/2015/white eye bird/D11-148.2	62.1
	8e	149	161.1	Avastrovirus	KT946721	AAstV/HKG/2015/white eye bird/D11-148.2	64.2
BR9	9a	2659	3935	Unclassified	KX884564	FAstV/CHN/2014/shrimp/BHWZXX13371	58.3
	9b	261	286.8	Avastrovirus	KT946721	AAstV/HKG/2015/white eye bird/D11-148.2	63.6
	9c	105	120.1	Avastrovirus	KT946721	AAstV/HKG/2015/white eye bird/D11-148.2	63.1
	9d	24	38.6	Mamastrovirus	KY575664	MAstV/MDG/2014/bat/UADBA SMG18894	68.4
BR 10	10a	40,150	6633.5	Avastrovirus	MK189093	AAstV/BRA/2010/ruddy turnstone/RT	66.7
BR 11	11a	64,214	67,410.2	Unclassified	KX884564	FAstV/CHN/2014/shrimp/BHWZXX13371	58.2
BR 12	12a	41,948	6639	Unclassified	KX884564	FAstV/CHN/2014/shrimp/BHWZXX13371	58.2
	12b	17	49.4	Avastrovirus	HM029238	AAstV/CHN/2009/chicken/Sichuan	75.0
BR 13	13a	723	93.1	Avastrovirus	KT946721	AAstV/HKG/2015/white eye bird/D11-148.2	64.7
	13b	72	100.2	Avastrovirus	KY320405	AAstV/SWE/2014/mallard/313	80.2
	13c	50	52.8	Avastrovirus	KT946721	AAstV/HKG/2015/white eye bird/D11-148.2	63.5

MastV = AstV identified in mammalian host; AAstV = AstV identified in avian host; RastV = AstV identified in reptilian host; FastV = AstV identified in fish and marine animals.

## Data Availability

Not applicable.
